# Genetic and Chemical Capsid Modifications of Adenovirus Vectors to Modulate Vector–Host Interactions

**DOI:** 10.3390/v13071300

**Published:** 2021-07-02

**Authors:** Denice Weklak, Daniel Pembaur, Georgia Koukou, Franziska Jönsson, Claudia Hagedorn, Florian Kreppel

**Affiliations:** Chair of Biochemistry and Molecular Medicine, Center for Biomedical Education and Research (ZBAF), Witten/Herdecke University, Stockumer Street 10, 58453 Witten, Germany; denice.weklak@uni-wh.de (D.W.); daniel.pembaur@uni-wh.de (D.P.); georgia.koukou@uni-wh.de (G.K.); franziska.joensson@uni-wh.de (F.J.); claudia.hagedorn@uni-wh.de (C.H.)

**Keywords:** adenovirus, capsid modification, chemical modification, PEGylation, HPMA, COVID-19 vaccine

## Abstract

Adenovirus-based vectors are playing an important role as efficacious genetic vaccines to fight the current COVID-19 pandemic. Furthermore, they have an enormous potential as oncolytic vectors for virotherapy and as vectors for classic gene therapy. However, numerous vector–host interactions on a cellular and noncellular level, including specific components of the immune system, must be modulated in order to generate safe and efficacious vectors for virotherapy or classic gene therapy. Importantly, the current widespread use of Ad vectors as vaccines against COVID-19 will induce antivector immunity in many humans. This requires the development of strategies and techniques to enable Ad-based vectors to evade pre-existing immunity. In this review article, we discuss the current status of genetic and chemical capsid modifications as means to modulate the vector–host interactions of Ad-based vectors.

## 1. Introduction

Adenovirus (Ad) vectors are the most frequently used vectors in gene therapy trials [https://a873679.fmphost.com/fmi/webd/GTCT, accessed on 30 June 2021] and are currently mainly applied in cancer therapies (reviewed in [1]) and as novel vaccines (reviewed in [2]). During the ongoing global SARS-CoV2 pandemic, Ad-based vaccines were successfully tested in clinical trials, were approved as vaccines against COVID-19, and are being used as efficacious preventive vaccines [3,4,5,6].

The human Ad has a ~38 kb double-stranded DNA genome. The viral capsid is composed of three major capsid proteins (hexon, penton base, and fiber) and four minor proteins (IIIa, VI, VIII, and IX) that are organized in icosahedral symmetry. Of these major capsid proteins, hexon is both the largest and most abundant protein of the shell. Twelve hexon homotrimers form one capsid facet, of which 20 are present in the capsid. Penton consists of homopentamers of penton-base proteins located on each icosahedral edge (vertex) forming the base for the vertex’s spike that consists of trimers of glycosylated fiber protein [7,8,9].

The recent success of Ad vectors in clinical trials is based on numerous advantageous features: Ad-based vectors exhibit broad tropism profiles, have large packaging capacities (up to ~36 kb), and persist episomally in infected cells. They can efficiently infect both dividing and quiescent cells, and the availability of scalable production systems enables large-scale vector production to high titers. However, despite these advantages the clinical use of Ad-based vectors in virotherapy and gene therapy still faces several hurdles. While local delivery, e.g., for genetic vaccination, is comparably unproblematic, systemic delivery of Ad bears some challenges mainly associated with widely pre-existing immunity, strong innate immune responses to the viral capsid, and robust adaptive immune responses to de novo synthesized viral and transgene gene products [10].

Upon systemic delivery, innate responses can occur within minutes to hours, leading to low systolic blood pressure, release of proinflammatory cytokines, thrombocytopenia, and transient liver toxicity. These adverse effects are triggered by interactions of vector particles with endothelial cells of the liver and vascular system, hepatocytes, Kupffer cells, platelets, macrophages, and dendritic cells (DC) [11]. Once in the blood stream, coagulation factor X (FX) binds to the hexon protein of the viral capsid [12], thus activating Toll-like receptor 4 on splenic macrophages resulting in a rapid clearance of vector particles from the spleen [13]. These innate immune responses are also observed for biologically inactive viral particles [14] or after administration of helper-dependent (HD) Ad vectors lacking all viral genes [15]. Therefore, innate immune responses seem to be largely independent from the expression of viral genes and instead relate to the viral capsid proteins themselves. Besides binding to coagulation factors, Ad capsid proteins are also bound by proteins of the complement system, such as C3 and C4BP, as well as natural immunoglobulin (IgM) antibodies. These interactions redirect vector particles from the blood stream to the liver [12,16] and mediate clearance through particle uptake by Kupffer cells [17,18].

The vector–host interactions of Ad known thus far are well described and build a basis for different capsid modification strategies. Mutating or blocking Ad vector interactions with cellular or noncellular host components is an efficient tool to detarget Ad from undesired off-target tissues. Moreover, safe and efficient therapeutic applications of Ad vectors require targeted cell entry opposing the broad tropism of adenoviruses. Genetic and chemical strategies that modify the interaction spectra of Ad capsid proteins may be used to retarget Ad vectors to new target cells or tissues.

Furthermore, pre-existing antibodies [19,20], originating either from previous Ad infections or vaccinations, can prevent the vectors from reaching target cells. For example, homologous prime-boost regimes using Ad-based vaccines, as applied in the recent pandemic [3], may elicit potent neutralizing antibody responses directed not only against the transgene itself but also against the vector. During boost vaccination, capsid-bound antibodies mediate vector sequestration by Fc receptor (FcR)-positive cells including DCs [21], neutrophils [22], and tissue-specific macrophages [23]. Antibody–vector complexes may further trigger the release of proinflammatory cytokines in macrophages by activating the intracellular antibody receptor TRIM21 [24,25,26]. Here, heterologous prime-boost regimes based on either different vector species [5,6] or platforms [27] could contribute to overcome this hurdle. However, with an emerging role of Ad-based vectors in vaccination strategies or virotherapy, these strategies will only postpone the initial problem. In the long-term, capsid modification strategies that shield vector particles from both innate and adaptive immune recognition may help to overcome this problem.

The above-described vector–host interactions and the challenges arising thereof highlight the necessity of viral capsid modifications in vector development to eliminate these obstacles. Since the majority of these interactions are interactions of the viral capsid with cellular and noncellular blood components, most strategies focus on modifications of the viral capsid, including genetic and chemical capsid modification approaches.

In the following, we will describe several mechanisms of vector detargeting to prevent unwanted interactions, vector targeting to direct vector particles to specific cell types, and combinations thereof.

## 2. Genetic Capsid Modifications

**Figure 1:**
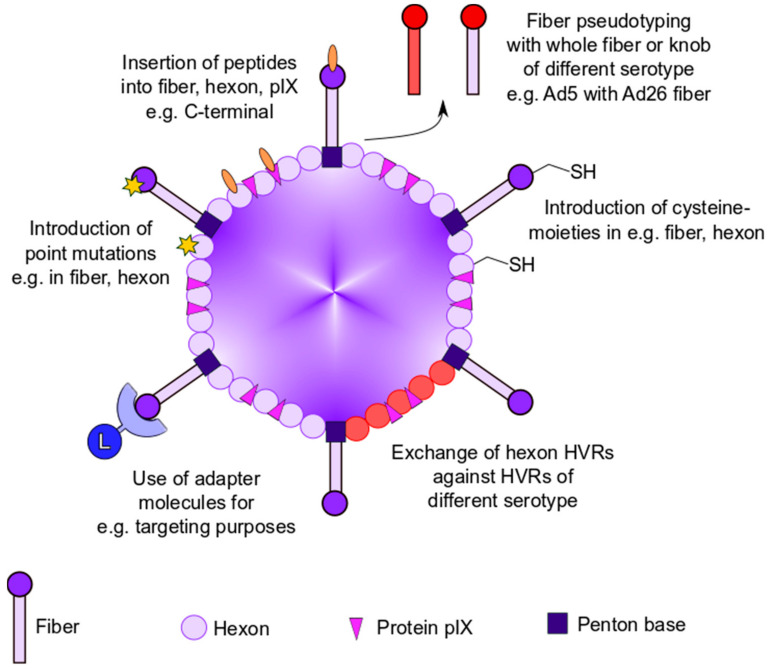
Overview of genetic modification strategies to modulate vector–host interaction. Genetic modification strategies for ablating viral tropism and anti-Ad immune responses by introduction of point mutations, insertion of peptides into capsid proteins, fiber pseudotyping, use of adapter molecules, or exchange of hexon hypervariable regions (HVRs).

Genetic modification of capsid proteins is the most direct approach to modulate immune recognition and Ad tropism. This can be achieved by either the introduction of mutations into the Ad capsid proteins themselves or by the incorporation of foreign peptides into the capsid. To date, all Ad capsid proteins of the outer capsid surface have been genetically modified. This part of the review focuses on previously described genetic modification strategies for abrogation of Ad liver tropism and anti-Ad immune response (Figure 1).

### 2.1. Genetic Modifications to Ablate Vector Tropism

The targeted cell entry of Ad is mediated by two fundamental steps. The first step includes the high-affinity attachment of the fiber protein to its primary receptor, the coxsackie and Ad receptor (CAR) [28]. This binding increases the proximity between virus and cell surface and allows for the binding of cellular integrins such as αvβ3 and αvβ5 to an Arg-Gly-Asp (RGD) motif in the Ad penton base [29]. The penton RGD motif is exposed by bending of the fiber protein due to a flexible joint in the shaft domain in a process previously described as ‘virus yoga’ (reviewed in [30]). The Ad-integrin interaction promotes the internalization of viral particles by clathrin-mediated endocytosis [31]. After partial disassembly of the viral capsid in the endosome and release of virus into the cytoplasm [32], replication-defective vectors transport their genome into the nucleus [33].

Importantly, while CAR is expressed in some therapeutically relevant cell types, it is also expressed in human erythrocytes, which also express complement receptor 1 (CR1). Therefore, human erythrocytes influence blood circulation and infectivity of systemically delivered viral particles [34]. However, as erythrocytes do not express integrins, Ad vector particles can bind to erythroycytes but cannot be internalized, indicating that this interaction is reversible [35]. A feasible approach to detarget Ad vecors from CAR-expressing cells is the introduction of specific point mutations into the fiber protein. Kirby et al. proved abolished binding to CAR for Ad5 vectors bearing a S408E, P409K, T477A, or L485K point mutation in their fiber protein [36]. However, the binding of CAR-ablated vectors to erythrocytes was not prevented, as antibody-mediated attachment to CR1 still occurred [37]. Moreover, blocking fiber-CAR interactions did not prevent liver sequestration, as the transduction of hepatocytes was still mediated by blood components bound to capsid proteins other than fiber [38]. The high-affinity binding of blood coagulation factors such as FX and IX to hexon leads to the sequestration of virus in the liver, possibly hindering efficient transduction of target cells [39]. FX binds Ad5 in the presence of Ca^2+^ and Mg^2+^ with picomolar affinity and increases heparan-sulfate-proteoglycan-mediated uptake into hepatocytes [12,39,40,41]. These observations demonstrate that although most detargeting approaches are straightforward, the interactions of Ad in vivo are far more complex and need to be better understood and carefully addressed to increase the safety and efficacy of Ad vectors in vaccinations and virotherapy.

#### 2.1.1. Generation of Chimeric Ad Vectors by Fiber Pseudotyping

An approach for modifying Ad tropism is the generation of chimeric Ad vectors using fiber pseudotyping (Figure 1). Fiber pseudotyping describes the substitution of the knob domain or the entire fiber protein with its counterpart of other serotypes. While CAR is used as the primary receptor for cell entry by most Ad types, interactions with other attachment receptors, such as CD46 (e.g., Ad21, Ad35), desmoglein-2 (e.g., Ad3), GD1a, and sialic acid (e.g., Ad37), have been described (reviewed in [42]). As such, fiber pseudotyping may be used to trim CAR-dependent serotypes to different cell types. This particular method of altering viral tropism was established by Krasnykh et al., and since its inception in 1996, many different chimeric Ad vectors with a broad spectrum of infection profiles have been developed [43]. For example, Shayakhmetov et al. demonstrated that a chimeric Ad5/35 vector showed increased infection of CD34^+^ hematopoietic cells [44]. In another example, the generation of an Ad5/3 chimera improved the infectivity of ovarian cancer cell lines allowing for the establishment of a retargeted oncolytic Ad [45,46]. Last but not least, in vivo experiments with pseudotyped Ad vectors uncovered a role of the fiber protein in Ad-induced thrombocytopenia. Mice infected with either wildtype Ad5 or Ad3 displayed a drop in platelet counts 2 days post infection. In contrast, the pseudotyping of Ad5 with either the complete fiber or fiber shaft of Ad3 prevented thrombocytopenia in infected mice [47]. These results indicate a role of fiber in Ad-induced thrombocytopenia that might be overcome by fiber-pseudotyping strategies. Importantly, while adverse blood clotting side effects have been observed for example with the chimpanzee Ad-vector-based COVID-19 vaccine, it is too early to speculate on the role of Ad fiber in this specific context since other factors such as the spike protein itself or fragments thereof could likely contribute to these rare observed events.

#### 2.1.2. Insertion of Targeting Peptides into Ad Capsid Proteins

One of the most frequently applied method of altering Ad tropism is the incorporation of foreign peptides into Ad capsid proteins to either increase or shift target cell infection. Most suitable for the incorporation of these foreign ligands is the fiber protein (Figure 1). While the C-terminus presents itself as an obvious location for peptide insertion, another optimal location has been identified as a solvent exposed, flexible loop between β-sheets H and I, the so called HI loop [48]. Peptides with a length of nearly 90 amino acids were C-terminally incorporated, while a peptide length of 83 amino acids was reported for the HI loop insertion site [49,50]. Commonly, an RGD-peptide is incorporated at the HI loop to increase integrin-based interactions between virus and host cells allowing for higher transduction efficacy of cancer cell lines, a strategy pioneered by the research group around *D.T. Curiel* [51]. Combining the insertion of an RGD-peptide in the HI loop with the insertion of a heparan sulfate binding polylysine in the C-terminus amplified the transduction efficacy of CAR-positive and -negative cells compared to un- or singly modified vectors [52]. Moreover, Curiel et al. reported that these vectors demonstrated a loss of natural CAR binding and infected ovarian carcinoma cell lines with increased efficacy [53]. As such, the safety and efficacy of RGD-incorporated Ad vectors was tested in clinical phase I trials for the treatment of ovarian cancer patients [54,55]. MacLeod et al. generated an Ad5 vector in which the epidermal growth factor (EGF)-like domain of heregulin-α was inserted into the HI loop to increase the in vitro infection of HER3/ErbB3- and HER4/ErbB4-expressing breast cancer cells [56]. It should be noted that incorporation of foreign peptides into the fiber protein can interfere with its trimerization and ultimately prevent viral assembly. Thus, the insertion site as well as the length of the desired targeting ligand should be considered carefully and will differ between different Ad types [57].

Another possible peptide insertion site is the cement protein IX which can be modified at its surface-exposed C-terminus. The incorporation of a polylysine sequence with an upstream FLAG peptide allowed for increased heparan sulfate binding and knob-independent infection [58]. Furthermore, IX is suitable for modification with large polypeptides and proteins, as the incorporation of enhanced green fluorescent protein (EGFP) with a length of nearly 240 amino acids was demonstrated, and only minimal effects on thermostability and bioactivity were observed [59]. Therefore, they demonstrated that protein IX has a higher peptide insertion capacity than the fiber protein.

In addition to protein IX and fiber, ligand insertion was also reported for the Ad hexon protein. It could be proven that hexon hypervariable regions (HVR) 2, HVR3, HVR5, HVR6, and HVR7 were appropriate for peptide insertion after testing feasibility with His_6_ epitopes [60]. While being rather limited in the length of the incorporated peptide, an example of hexon HVR5 accommodating a foreign RGD-peptide showed increased transduction of human vascular smooth muscle cells with only minor effects on virus viability and growth [61]. Moreover, the introduction of point mutations in the Ad hexon protein presents an elegant solution for escaping blood coagulation factor-mediated hepatocyte tropism. Doronin et al. demonstrated that a point mutation (T425A) within HVR7 of hexon abolished binding of FX to Ad5 and reduced hepatocyte infection [13]. Of note is that interfering with FX association can lead to natural IgM- and complement-mediated neutralization of Ad [62].

#### 2.1.3. Adapter-Based Strategies to Ablate Ad Tropism

A different strategy for employing altered viral tropism is the use of adapters serving as fusion proteins between capsid structure proteins and cell-binding ligands (Figure 1). One of these adapter strategies utilized the fusion of single chain variable fragments (scFv) to peptides such as EGF to target EGF-receptor expressing cells, thus enhancing the efficacy of Ad gene therapy [63]. In a similar way, Barry et al. fused the hexon-binding domain of FX to scFv to retarget Ad vectors to cells expressing Her2, ATP-binding cassette protein G2, and EGF-receptor [64]. Parrott et al. also reported the use of another adapter strategy in which a biotin-acceptor peptide (BAP) was fused to the C-terminus of fiber. This peptide is metabolically biotinylated in producer cell lines and can be used for retargeting purposes by conjugation to biotinylated antibodies [50]. However, fusing BAP to protein IX failed retargeting when biotinylated antibodies were used, and retargeting was only achieved when biotinylated ligands instead of antibodies were used [50,65]. An alternative method was reported by Dimitriev et al. in which a soluble, truncated version of CAR was fused to EGF, resulting in an increased infection of EGF-receptor overexpressing cancer cell lines compared to untargeted Ad or EGF-receptor negative cells in vitro [66]. These adapter strategies are based on noncovalent protein–protein interactions. Due to this, natural CAR, FX, or antibody binding can compete with the adapter molecule binding, ultimately preventing vector retargeting.

### 2.2. Genetic Modifications to Circumvent Anti-Ad Immune Responses

Another difficulty of systemic delivery of Ad vectors is the high prevalence of anti-Ad immunity in the population. Neutralizing antibodies against common Ad types, such as Ad5, is relevant, and means of immune escape must be taken into consideration prior systemic delivery of Ad vectors, especially in regard to the current coronavirus pandemic. As mentioned above, worldwide vaccination with Ad-vector-based vaccines such as Vaxzevria^®^ (AstraZeneca plc) and *Ad26.COV2-S* (Janssen Pharmaceutica N. V.) will increase the prevalence of neutralizing anti-Ad antibodies in the population and probably limit the efficacy of subsequent treatment with Ad vectors, including prime-boost regimens. The combinatorial utilization of different de- and retargeting strategies described above requires a detailed understanding of the interactions not only between Ad capsid proteins and target cells but also between Ad and (noncellular) blood components. For example, while the binding of FX to Ad hexon increases the transduction efficiency of hepatocytes, it also acts as a shield and thus prevents the binding of natural or pre-existing antibodies. Therefore, the ablation of FX binding (e.g., by introduction of point mutations in hexon HVR7) may result in increased IgM- and complement-mediated vector neutralization after systemic delivery [62].

As most of the neutralizing antibodies are directed against HVRs of Ad hexon [67], the generation of chimeric Ad variants by replacing these regions against HVRs of a less prevalent serotype is one method to hinder antibody-mediated neutralization (Figure 1). For example, Roberts et al. demonstrated that the exchange of all seven HVRs of Ad5 hexon for their Ad48 counterparts increased Ad immune escape in vivo [67]. However, replacing HVRs proved to be insufficient for anti-Ad immune escape [68]. Still, it should be noted that Roberts et al. reported a three- to fivefold lower production yield in comparison to the parental Ad5 vectors. In addition, most of their chimeric vectors were nonviable, indicating structural and biochemical constraints in regard to the manipulation of hexon HVRs [67]. In a different approach, Rojas et al. incorporated an albumin-binding peptide into hexon for shielding via binding to blood serum albumin after systemic delivery [69]. While it was shown that the virus was protected from neutralization by natural antibodies, Rojas et al. observed a decrease in the transduction efficacy of the albumin-binding vector [69]. Recently, another method for Ad escape from natural IgM- and complement-mediated neutralization has been reported. Atasheva et al. demonstrated that the deletion of a small stretch of negatively charged amino acids in the HVR1 in combination with an FX-ablating mutation could prevent the binding of natural IgM antibodies and complement components [70]. In summary, the research group generated an Ad vector that resisted inactivation by blood factors and liver cell sequestration and did not trigger hepatotoxicity. This is, to our knowledge, the most complete approach to genetic capsid modifications with substantial modulation of Ad vector–host interactions so far. However, the interaction of anti-Ad antibodies with the vector after systemic delivery requires further research.

#### 2.2.1. Removal of Viral Genes from the Vector Genome

Ad-associated immunogenicity and toxicity can be decreased by the use of high-capacity (HC) or helper-dependent (HD) vectors, also termed ‘gutless vectors’. HD-Ad vectors were generated by the deletion of all viral coding sequences but not the inverted terminal repeats (ITR) necessary for genome replication and the packaging signal (Ψ) (reviewed in [71,72]). The deletion of nearly all viral genes allows for a high cloning capacity of about 36 kilobases (kb). However, efficient DNA packaging requires a minimum vector size of 27 kb, and as such, stuffer DNA is required to reach minimum viral packaging size [73]. While the first DNA stuffers relied on the use of lambda phage, bacterial, or yeast DNA, the use of intronic sequences of human genes, such as hypoxanthine-guanine phosphoribosyltransferase (HPRT), has become more frequent. HD-Ad vectors containing lambda-phage stuffer DNA showed limited gene expression as well as an increased cytotoxic T cell (CTL) response in vivo [74], while vectors equipped with HPRT-derived stuffer DNA displayed prolonged transgene expression without inducing CTL responses [74]. Since the dsDNA itself may trigger intracellular innate immune responses via TLR signaling [75], stuffer DNA sequences should be designed carefully.

With all viral genes being deleted, HD-Ad vectors rely on a helper Ad supplying viral proteins in trans for propagation (reviewed in [71]). This risks contamination of, e.g., gene therapy preparations with helper Ad and replication-competent particles (reviewed in [76]). Due to the lack of viral gene expression, HD-Ad exhibit prolonged transgene expression and reduced toxicity. While the adaptive immune response to these vectors is dampened, acute inflammatory responses were still observed [15]. Despite this, HD-Ad vectors were successfully used for long-term expression of transgenes in nonhuman primates [77,78].

#### 2.2.2. Geneti-Chemical Modification Strategies

**Figure 2:**
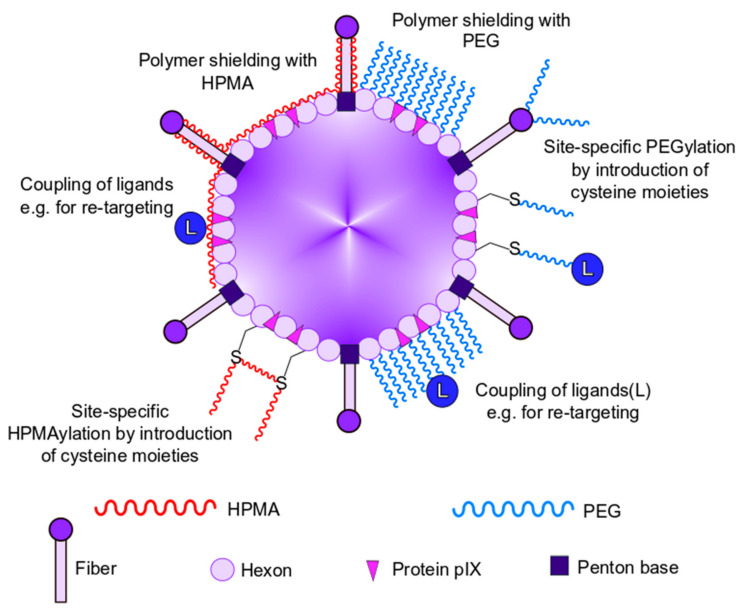
Overview of chemical modification strategies to modulate vector–host interaction. Chemical modification strategies for polymer capsid shielding with polyethylene glycol (PEG) or poly-*N*-(2-hydroxypropyl) methacrylamide (HMPA) and retargeting purposes by use of ligands.

Finally, another approach to prevent liver cell sequestration and antibody-mediated neutralization of Ad is the genetic introduction of cysteine moieties into Ad capsid proteins, allowing for site-specific chemical coupling of polymers (Figure 1 and Figure 2) [79]. This so-called geneti-chemical approach enables researchers to shield specific capsid proteins from unwanted interactions, to attach ligand molecules to the capsid, and to study vector–host interactions after capsid modification in a position-dependent manner. As an example of this geneti-chemical modification approach, Prill et al. introduced a cysteine moiety in hexon HVR5 which allowed for chemical coupling of small maleimide-activated polyethylene glycol moieties, leading to a reduction in hepatocyte transduction after systemic delivery [80]. The incorporation of foreign cysteine moieties is not limited to the hexon protein, as modulation of the fiber HI loop and protein IX has also been shown to be successful [79,81]. Generally, the chemical and geneti-chemical coupling of polymers to various capsid proteins are promising approaches to evade anti-Ad immune responses and will be discussed in more detail in the following parts of this review.

All things considered, engineering vectors for anti-Ad immune escape by genetic modifications alone may prove difficult. HD-Ad vectors still elicit innate immune responses, while the exchange of hexon HVRs affects vector viability, and shielding with albumin reduces viral infectivity. The generation of chimeras between Ad types or with different viruses (e.g., by inserting the genome of small viruses into the Ad genome) may present itself as a future endeavor for escaping anti-Ad immunity as well as for improving target cell transduction efficacy and biodistribution (reviewed in [76]).

Conclusively, genetic modifications of the Ad genome offer a robust toolkit for altering Ad tropism and may provide means of escaping anti-Ad immunity. While the possibilities seem nearly endless, the combination of different re- and detargeting approaches requires an excessive amount of knowledge about the interaction of Ad and blood components. It has been shown that the incorporation of peptides as well as the generation of chimeric Ad vectors can be rather challenging. Interference with Ad structural integrity can result in nonviable, less infectious vectors, and thus the choice of motif and site of insertion should be considered carefully. Furthermore, Ad-based vaccines are currently employed in the ongoing SARS-CoV2 pandemic, and therefore genetic modifications alone may be insufficient to prevent anti-Ad-mediated neutralization. Chemical modification of the Ad capsid by polymer shielding could provide additional help for the evasion of anti-Ad immunity.

## 3. Chemical Shielding of Ad Vectors

As mentioned above, genetic modifications of Ad vectors were used to evade from the immune system, to detarget particles from their natural tropism, and to retarget them to new receptors [82]. However, genetic modifications are limited by several obstacles, including structural capsid protein integrity and stability often in conjunction with impaired vector production [60]. In contrast, the chemical modification of the vector capsid ensures (i) a highly efficient vector production without impairment by protein structural changes, (ii) a comparably fast and easy capsid modification strategy, (iii) no safety concernsdue to inherited vector immune evasion properties, and (iv) heterogeneous capsid modifications given by a wide variety of appropriate molecules that allow for the chemical modification of the capsid under mild conditions [83]. These molecules are, in general, strongly hydrophilic polymers that are activated with amine- or thiol-reactive groups to bind to α- or ε-amino groups of the N-terminus and of lysine residues, respectively. In the case of thiol-reactive groups, these molecules react with reduced cysteine residues (Figure 2). Besides the possibility to define the binding site of the polymer, the binding mode can be also selected. Thereby, the type of reactive group determines whether the binding of the polymer is irreversible or bioresponsive, i.e., reversible under certain cellular conditions (e.g., reducing or acidic compartments) [84,85]. Bioresponsive modifications offer the option of evading immune components in the blood while ensuring a regular intracellular trafficking of the vectors by uncovering polymer modified proteins after cell entry. Further variety of the polymers is offered by the number of reactive groups per polymer. A polymer that possesses only one reactive group would protrude from the capsid whereas a polymer with multiple reactive groups would closely attach to the capsid surface (Figure 2). Thus, even the size of the vector capsid could be modified by the choice of the polymer, which could have the potential of altering vector tropism [82,86]. Chemical modification of vector capsids can be performed very fast (about 1 h) and under mild conditions (room temperature, physiological pH) and, furthermore, without impairment of vector production. Moreover, it offers endless options that allow for detargeting and even retargeting of the vectors by the introduction of new ligand binding sites. Here, we discuss key results from the two major polymers polyethylene glycol (PEG) and poly-*N*-(2-hydroxypropyl) methacrylamide (HPMA), which have successfully been used for chemical modification of Ad vector capsids for protection against immune and blood factors as well as for de- and retargeting (Table 1).

## 4. Shielding with HPMA

### 4.1. HPMA-Coating of Ad

HPMA is one of the important polymers to chemically modify the Ad capsid. The main advantage of HPMA polymers is their high biocompatibility and the low immunogenicity which were tested for a wide variety of different HPMA polymers (reviewed in [87]). Over two months, in vivo assays in mice showed no significant influence of the basic immune defense activity after treatment with a 25 kDa HPMA and a high dose of 2 g HPMA per kg body weight [88]. The tested basic immune activities included humoral immune responses, activation of complement pathways, phagocytotic activity, and bone marrow status. Furthermore, the examined doses of 2 g HPMA per kg body weight were much higher than the doses that would be used in clinical practice. Moreover, the size of the administered HPMA polymer was shown to be decisive regarding their immunogenicity [89]. Říhová et al. detected a two- to fivefold increase of the number of antibody-forming cells in the spleen in mice after injection of 150–200 kDa HPMA polymers in comparison to 5 kDa HPMA polymers [89].

Evasion from undesired vector–host interactions of Ad vectors can be achieved by modifying the vector capsid with diverse HPMA polymers and thus limit vector sequestration and reduce vector immunogenicity. One of the first attempts was presented in 2001 by Fisher et al. with an all-in-one approach [90]. In brief, an Ad type 5 vector was chemically modified with an amine-directed HPMA that was activated with ONp groups. An efficient coupling of the polymer to the vector was shown, and in vitro transduction assays with A549 cells revealed a significant detargeting of the shielded vectors from these CAR-positive cells. Furthermore, the coupling of fibroblast growth factor (FGF) or vascular endothelial growth factor (VEGF) to the HPMA-coated vectors resulted in a retargeting towards receptor-positive cells in vitro. Additionally, an enzyme-linked immunosorbent assay (ELISA) showed protection against neutralizing antibodies [90]. This all-in-one approach was a door opener to the field of HPMA-coated Ad vectors.

### 4.2. Chemistry of HPMA Coupling

The coupling of HPMA polymers can be generally performed at amine groups or genetically introduced thiol groups of solvent accessible residues on the Ad capsid surface (Figure 2). The conditions of HPMA coupling were reported to be very mild with a pH of 7.2–7.8 and at room temperature [84,90,91]. As reviewed by Říhová et al., another welcome feature of HPMA polymers is the low immunogenicity of the molecule [87]. HPMA polymers used for Ad capsid modification possess multiple reactive side chains that bind cooperatively to multiple sites at the capsid surface [92]. Kreppel and Kochanek reviewed that this multivalent reaction mode closely attached HPMA to the capsid surface, which is in contrast to the binding of monovalent PEGs which protrude with one free end from the capsid surface (see Figure 2) [83]. Thus, the size of HPMA-modified vectors increased only slightly when compared to the molecular weight of the used polymer, which commonly ranges from 12 to 150 kDa [34,84,90,91,93,94]. However, it was shown that by altering the initial concentration and the reaction temperature during polymer synthesis, the molecular weight of HPMA copolymers could be influenced [93]. In the following, we give an overview of the different coupling strategies using HPMA, and we further describe the chemical properties of HPMA polymers.

#### 4.2.1. Amine-Directed HPMA Coupling

**Figure 3:**
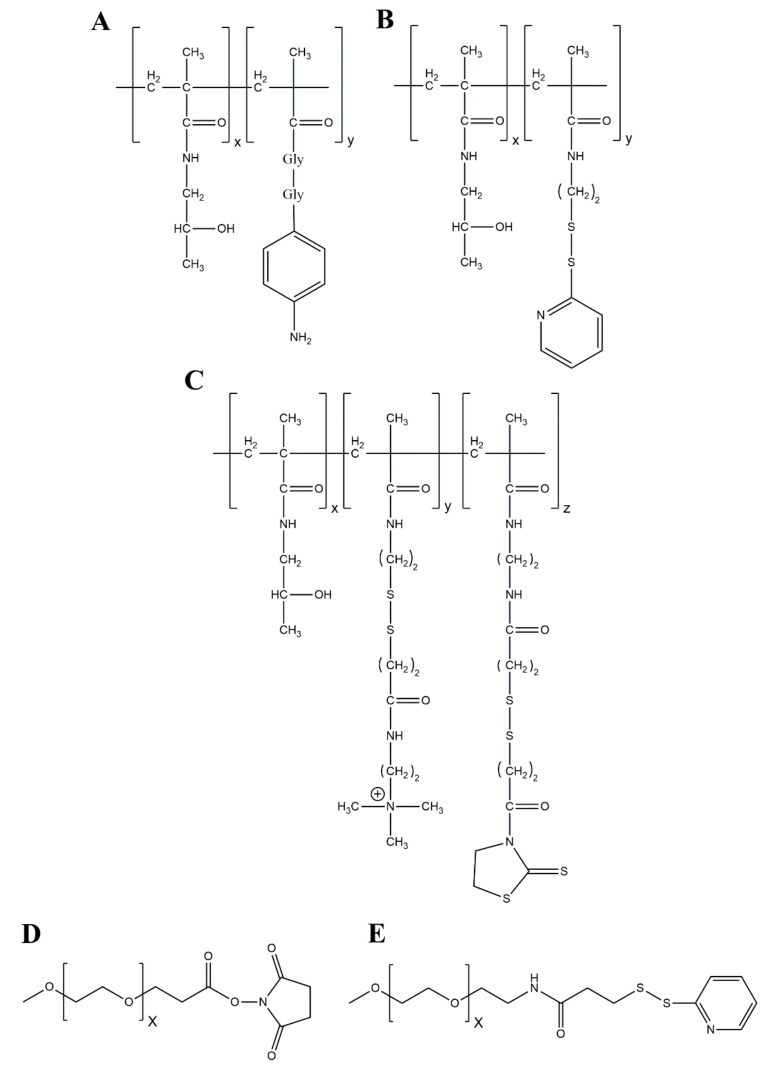
HPMA copolymer and PEG structures. (**A**) HPMA copolymer activated with a diglycine-linked ONp as presented by Fisher et al. [90]. ONp as an amine-reactive group will covalently bind to ε-amino groups of lysine residues on the capsid surface. Commonly used ratio for the monomers is 9:1 (X:Y). (**B**) Orthopyridyl disulfide (OPSS)-activated HPMA as presented by Prill et al. [84]. This copolymer will bind to genetically introduced cysteines on the capsid surface and results in a disulfide bound (bioresponsive, reducible) HPMA-coated vector. (**C**) “Next generation HPMA copolymer” with disulfide linkers (bioresponsive, reducible) in the quaternary amine (QA) monomer and in the amine reactive carbonyl thiazolidine-2-thione (TT) monomer as presented by Subr et al. [91]. The amine reactive TT group will covalently attach the HPMA to ε-amino groups of lysine residues on the capsid surface, while the QA group will bind to the negatively charged surface of the capsid to enhance the efficacy of the covalent attachment. (**D**) Succinimidyl propionate (SPA)-activated polyethylene glycol (PEG). SPA is an amine-reactive group and will covalently and irreversibly bind to ε-amino groups of lysine residues on the capsid surface [97]. (**E**) OPSS-activated PEG. OPSS is a thiol-reactive group and will covalently, bioresponsively bind to genetically introduced cysteines [98].

The amine-directed HPMA copolymers that are mostly used in chemical Ad modification allow for the binding of the polymer to multiple, solvent-exposed terminal α-amino groups and ε-amino groups of lysine residues, respectively [93,94]. Thus, the multivalency of HPMA copolymers facilitates a cooperative binding to the Ad capsid that provides about 18,000 possible binding sites for amine-directed HPMA coupling [90,95]. It was reported by Green et al. that up to 90% of these primary amines could be modified by HPMA coating [96]. The HPMAs used, were activated by 4-nitrophenoxy (ONp) side chains that covalently attach the polymer to the vector capsid (Figure 3A). of the different amine reactive HPMA copolymers are presented in Figure 3.

#### 4.2.2. Thiol-Directed HPMA Coupling

By the genetic introduction of cysteines into vector capsid proteins, solvent-exposed thiol groups can be introduced into the Ad capsid surface. This approach allowed for thiol-directed HPMA coupling at introduced cysteines in the hexon protein [84]. In comparison to amine-directed HPMA coupling, this method enables a position-specific coupling of the polymer to selected capsid proteins [98]. For thiol-directed HPMA coupling, the HPMA polymers were activated with maleimide or orthopyridyl disulfide (OPSS) groups (Figure 3B) to generate irreversible or bioresponsive bonds between HPMA and vectors, respectively [84]. Further properties of the thiol-directed HPMA coupling are presented below.

#### 4.2.3. Next Generation HPMA Polymers—Bioresponsive HPMA Coupling

A wide diversity of HPMA copolymers can be synthesized by altering the reactive group or introducing charged side chains (e.g., quaternary amino groups). The choice of the type of the reactive group is crucial for the intracellular fate of the vector. As mentioned above, an activation of HPMAs by ONp side chains resulted in an irreversible covalent attachment of the polymer to the vector capsid (Figure 3A). As shown for HPMA-coated DNA/polyethyleneimine (PEI) complexes, the irreversible ONp-HPMA coating resulted in an ablation of transgene expression, while it could be restored with a disulfide-linked HPMA coating [99]. Based on these findings, Subr et al. presented the “next generation HPMA copolymers” that included a degradable disulfide bond in the spacer between the amine reactive carbonyl thiazolidine-2-thione group and the HPMA (which possessed additional quaternary amines for a higher coating efficiency, Figure 3C) [91]. Prill et al. reported with live cell imaging that an irreversible attachment of HPMA copolymers to specific sites on the hexon protein caused an intracellular trafficking impairment of the vectors [84]. Moreover, this trafficking impairment could be converted to a trafficking delay by using bioresponsive disulfide-bound HPMAs (Figure 3B). The authors could show that these biorepsonsive polymers can be shed from the particles in the reducing intracellular environment in a traceless manner, i.e., leaving no chemical residues on the capsid surface. Wang et al. reported that an electrostatic attachment of HPMAs to the vector capsid with biodegradable linkers enhanced transgene expression significantly compared to nonbiodegradable linkers [100]. Taken together, these results show that bioresponsive HPMAs are more suitable for chemical Ad vector modification, than the irreversible attached HPMAs. These results highlight the importance to maintain dynamic abilities of the vector particles after chemical modifications.

As mentioned above, the HPMAs presented by Subr et al. possessed quaternary amines (QA) as an additional modification of the HPMA copolymer that is not solely based on a chemically reactive group. The QAs imparted a positive charge to the HPMAs and enabled them to bind to the negative vector capsid surface, especially to the hexon protein [91]. Thus, they bind the vector capsid with higher affinity and at the same time convert the slightly negative surface (ζ) potential into a neutral or even minimal positive charge which—as discussed later (see Section 4.3)—may alter the vector tropism by the binding of the positively charged vector capsid to negatively charged surface molecules [34,84,100].

Another feature of vector-attached HPMAs is the excess of reactive groups which did not react with the capsid. By the addition of ligands with suitable binding sites (e.g., antibodies, growth factors), these ligands could be adhered to the HPMA-vector complex, allowing an easy retargeting of the whole complex [90,101]. Alternatively, the ligand could react with the HPMA prior to the coating of the vector capsid [102].

### 4.3. Detargeting and Immune Evasion of HPMAylated Ad Vectors

Intravenous delivery of Ad vectors remains a favorable route for virotherapy. In that case, several barriers must be overcome, such as rapid inactivation of the vectors by humoral and cellular immune and blood components. The wide range of cell types targeted by the wildtype Ad further leads to off-targeting in tumor therapy [103]. The intraperitoneal treatment of mice with Ad vectors was shown to result in peritoneal adhesion formation and bowel obstruction, likely caused by inflammatory effects that could be circumvented by altering the vector tropism with HPMA coating [102].

HPMA-modified vector capsids were shown to evade humoral immune and blood factors as well as the CAR- and FX-mediated binding to host cells. Subr et al. assessed three different amine reactive HPMA copolymers and demonstrated in vitro that exhaustive coating of Ad vectors greatly reduced CAR-mediated transduction and binding to human erythrocytes, respectively [91]. In addition, they demonstrated an ablation of FX-mediated transduction in in vitro experiments for the vectors modified with QA containing HPMAs and, moreover, a great reduction of CR1-mediated binding to human erythrocytes. These results indicated an ablation of the natural Ad tropism for the QA-containing HPMA-modified vectors and, likewise, partial protection against the complement and binding of antibodies. Green et al. showed that in in vivo experiments with BALB/c mice, Ad5 vectors modified with a 31.6 kDa thiazolidine-2-thione (9.5 mol%)-activated HPMA had an extended plasma circulation time compared to unmodified Ad5 and Ad11 [96]. Since Ad11 is unable to infect the cells of BALB/c mice (including hepatocytes) the extended circulation time of HPMA-modified vectors was therefore not only a result of an ablation of the natural tropism, but also due to a prevention of phagocytosis by macrophages. Furthermore, for the HPMA-coated vectors, Green et al. demonstrated a stable circulation for 30 min in BALB/c and melanoma tumor-bearing C57BL/6 mice after removal of Kupffer cells by preadministration of clodronate liposomes. In fact, Green et al. were able to show a complete prevention of tumor growth in HEPG2 xenograft-bearing mice by a 29-day treatment of HPMA-coated wildtype Ad5 and clodronate liposomes [96]. This extended plasma circulation time was crucial for a passive targeting of the vectors to the tumors, which was described by the enhanced permeability and retention (EPR) effect in the tumor area [104]. Fisher et al. first described that the EPR effect can also be exploited by using an ONp-HPMA-modified Ad5 vector and showed a targeting of subcutaneous AB22 mesothelioma tumors in BALB/c mice. Additionally, the initial vector dose found in the liver was greatly reduced compared to uncoated Ad5 vectors. Nevertheless, specific transgene expression (expression per vector particle) of coated vectors in tumor was diminished compared to uncoated vectors, and it was postulated that this might be caused by an altered infection pathway [92].

The altered tropism of HPMA-coated Ad5 vectors was also assessed by Prill et al. with geneti-chemically modified vectors (as described by Kreppel et al. in [79]). In their study, the selective binding of maleimide- or OPSS-activated HPMAs to introduced cysteines in the hexon protein was used to maintain CAR-mediated transduction capacity but, at the same time, to shield against FX-mediated transduction [84]. This approach resulted in decreased liver tropism and thus reduced transgene expression in the liver of vectors modified with uncharged HPMA. In contrast, positively charged HPMA mimicked the effect of FX-mediated liver transduction, underlining the necessity of a careful HPMA design. Regardless of whether the vectors were modified with charged or uncharged HPMAs, the number of vector particles in the spleen, lung, and kidneys was markedly reduced compared to the number of unmodified vector particles [84]. Moreover, the plasma circulation time of coated vectors in comparison to unmodified vector particles increased significantly to 2.4-fold for an uncharged HPMA and 8.8-fold for a charged HPMA, respectively. The effect of positively charged HPMAs binding to negatively charged surface molecules (e.g., heparan sulfate proteoglycans, HSPGs) of cells was also described by Wang et al. [100].

Stevenson et al., who modified chicken embryonal lethal orphan virus (CELO, fowl Ad type 1) with HPMA, showed a reduced neutralization of coated human Ad5 vector particles in human serum, reflecting a partial evasion of the humoral immune defense by HPMA coating [105]. Furthermore, in a capture ELISA, Fisher et al. showed no detectable antibody binding with a fully HPMA-coated Ad5 vector [90]. Moreover, Carlisle et al. demonstrated an improved resistance against neutralizing antibodies and plasma components when using a retargeted Ad5 vector modified with bioresponsive QA-bearing HPMA. Additionally, an ablation of complement or fiber-mediated binding to erythrocytes was achieved [34]. However, the immune evasion of HPMA-coated Ad vectors in human serum is a major challenge that needs to be assessed with every new HPMA copolymer.

### 4.4. Retargeting of HPMAylated Ad Vectors

In addition to the passive tumor targeting of fully shielded Ad vectors, an active retargeting by the introduction of the appropriate ligands to the shielded vector capsid is a highly promising and versatile option for chemical Ad vector modification.

As described above, the excessive reactive groups of HPMA copolymers attached to Ad vectors could be used to introduce ligands. Fisher et al. detargeted HPMA-coated Ad5 vectors from CAR expressing cells, and after the introduction of fibroblast growth factor (FGF) or VEGF, vectors were retargeted to cells with corresponding receptors [90]. The FGF-retargeted vector was able to induce a 10-fold increase in transgene expression in SUIT-2 cells (pancreatic ductal carcinoma) in mice in vivo. The same retargeting strategy was used for a CELO virus with a successful retargeting in vitro [105]. However, in vivo, in BALB/c mice, a higher number of these vectors were found in all examined organs compared to unmodified Ad5 vectors. However, the transgene expression of the retargeted CELO vectors was observed to be greatly reduced. The authors stated that the high particle number in organs likely originates, on one hand, from a faster degradation of Ad5 compared to CELO vectors and, on the other hand, probably from the low-affinity binding of FGF to widely presented HSPGs. Nevertheless, the reduced transgene expression might be a result of an altered intracellular trafficking that was perhaps caused by differences between human and avian cells [105]. Importantly, targeting by FGF could lead to excessive binding to human erythrocytes and may therefore be unfavorable for systemic delivery [105].

EGF was also employed as a retargeting ligand [34,102]. Morrison et al. retargeted wildtype Ad5 to EGF receptor by coating the vector with thiazolidine-activated and EGF-bearing HPMAs. They showed detargeting from the CAR receptor and retargeting to the EGF receptor. The complex of EGF bound to its receptor was endocytosed, and the EGF still colocalized to its receptor in perinuclear areas. Furthermore, a murine xenograft model with intraperitoneal implanted SKOV-3 cells was used to demonstrate the efficacy of the retargeted viruses. The intraperitoneal injection of detargeted (HPMA-coated) virus showed no effect on survival, whereas the retargeted (EGF-HPMA-coated) virus and, in a similar way, the uncoated virus resulted in longer survival of the mice compared to the PBS control. However, fewer side effects (peritoneal adhesions and bowel formation) were observed for coated virus in comparison to uncoated virus [102]. Similar effects were reported for the same HPMA-coated vector when retargeted with the monoclonal antibody Cetuximab. Cetuximab is directed to the EGF receptor and is used for colorectal cancer therapy [101,106].

Another interesting approach for the retargeting of HPMA-coated Ad5 vectors was presented by Li et al. who used an activable cell penetrating peptide (ACPP) to retarget the coated vectors to matrix metalloproteinase (MMP)-overexpressing cells [107]. ACPP consists of a polycationic (polyarginine) domain, which is able to penetrate cell membranes and is linked via an MMP-cleavable linker to an inhibitory, polyanionic domain. The polyanionic domain can be cleaved in MMP-overexpressing tissues from the ACPP, allowing the polycationic domain to bind to and enter cells. Li et al. showed that the loss of transduction efficiency of a fully detargeted HPMA-coated vector could be regained by linkage of the ACPP to the HPMA-coated vector. Furthermore, reduced neutralization of the coated vector by neutralizing antibodies was shown. The selectivity of the ACPP retargeting was demonstrated by monitoring transgene expression in vitro. While human bronchial epithelial cells exhibited only diminished transgene expression, three assessed tumor cell lines showed significantly higher expression [107].

Conclusively, HPMA copolymers were shown to successfully and efficiently detarget Ad vectors from their natural target cells. More importantly, HPMA polymer shielding was able to protect the vectors against neutralizing blood components while introducing possibilities for vector retargeting by the introduction of appropriate ligands.

Nevertheless, HPMA coating must be designed carefully as it is able to coat the vector particles, resulting in a fully passive targeting that might not be sufficient for a successful tumor therapy [108]. In contrast, incomplete HPMA modification of the vectors that still ensures maintenance of the natural tropism by the binding of vector proteins to host cell receptors (e.g., fiber knob binding to CAR) likewise offers an unfavorable possibility of neutralization of the vectors by immune components [84]. Therefore, the most promising solution for vector design should combine both genetic and chemical modifications. While the polymer shielding is best for the repeated delivery of the vectors without triggering immune responses against the vector, genetical modification of the capsid could close the remaining gaps in the shielding or retargeting by polymers. This may also offer an option for a two-step targeting for oncolytic vectors. These vectors may lose their polymer coating upon transduction mediated by the first chemically introduced targeting molecule to present a second genetically introduced targeting molecule after replication in tumor cells [82].

## 5. Shielding with Polyethylene Glycol (PEG)

### 5.1. Chemistry of PEGylation

An alternative to HPMA is polyethylene glycol (PEG). PEG molecules are synthetic yet biocompatible compounds of repeating ethylene glycol units, widely used in cosmetic and pharmaceutical industries. Τhey are hydrophilic and can be produced in various lengths up to 40 kDa in branched or linear forms. PEG molecules are able to attach to proteins with covalent or noncovalent bonds. Due to their low toxicity and immunogenicity, the conjugated protein acquires new potentials such as an increased half-life and solubility when administered in vivo without loss of its function [109,110]. Thus, protein PEGylation has been proven, for many years now, to be a good and robust approach in the field of targeted drug delivery systems. To date, numerous drugs with PEGylated compounds have been approved. For example, PEGylated interferons have been used for therapies of multiple sclerosis [111] and hepatitis C [112]. Pegademase bovine has been applied in enzyme replacement therapy for severe combined immunodeficiency [113], while PEGylated peptides have been used for acromegaly [114,115].

The chemistry behind the coupling of PEG moieties to proteins is comparable to the coupling of HPMA moieties described above. Here, reactive groups such as maleimide (mal-) or succinimidyl propionate (SPA-) covalently bound to PEG molecules can couple such reactive PEG molecules to ε-amino groups of naturally occurring lysine residues or -SH groups of genetically introduced cysteine residues within the viral capsid. Additionally, linkers can contribute in the vector’s performance in vivo [83]. PEG molecules can be engineered as monofunctional moieties with one reactive group or as bifunctional moieties offering two different reactive groups at both ends. Bifunctional PEGs can be further considered as homobifunctional when the same reactive groups are used or heterobifunctional if these are different [83]. An overview of chemical modification strategies with PEG is shown in Figure 2. Since the structure and composition of the Ad capsid are described in great detail, the density of the covalently coupled PEG shield can easily be regulated by the number of PEG molecules used.

In general, PEGylation, such as HPMAylation, can be performed under mild conditions, such as room temperature and at a pH of 7.4–8.2 [83]. Since the PEGylation of therapeutic protein compounds has been shown to significantly reduce immunogenicity and antigenicity [116,117], coupling PEG molecules to viral capsid proteins might also contribute to viral vector gene therapy. By PEGylating the major capsid proteins fiber, penton and hexon, vectors can be shielded from various interactions with blood and immune components, thus overcoming the most prominent obstacles in their application in gene therapy.

One of the most pivotal attempts of chemical capsid modification via PEGylation was performed by O’ Riordan et al. in 1999 [118]. In fact, that was one of the very first attempts to chemically alter the Ad capsid structure. The authors engineered a whole capsid shield using covalently attached tresyl-monomethoxypolyethylene glycol (T-MPEG) molecules to ε-amino groups of capsid lysine residues in first generation Ad5 vectors. The T-MPEG-shielded vectors remained bioactive in comparison to cyanuric chloride-activated MPEG (CC-MPEG) or succinimidyl succinate MPEG (SS-MPEG) vectors, and, in in vitro studies, T-MPEG vectors reached higher gene expression levels in the presence of neutralizing antibodies. After intranasal immunization of mice with T-MPEG Ad2-βgal, MPEG Ad2-βgal, or unshielded Ad2-βgal, expression of the transgene product in the lung tissue cells of mice treated with T-MPEG vectors was highest, also indicating the efficient evasion of Ad neutralization in vivo due to this PEGylation strategy [118].

#### Bioresponsive PEGylation Approaches

While PEGylation protects Ad vectors from host immune and blood components (see Section 5.3), chemical modification of the capsid may interfere with the biological functionality of the vectors. Hence, the reactive group of a PEG molecule defines not only its reactivity towards functional groups on the vector capsid, but also the bioresponsiveness of the PEG–vector capsid bond. A well-designed reversible bond can thereby be bioresponsive, i.e., reversible under certain physiological conditions (e.g., in low pH in endosomes or in highly reducing cell compartments). The environmentally triggered release of the PEGs can restore the initial biological function of the capsid after delivery of the protected PEGylated vectors to the tissue of interest.

As described above, this concept was presented by Carlisle et al. for HPMA coated DNA–PEI complexes and was engineered with a reducible disulfide bond between HPMA and the DNA–PEI complex [99]. Moreover, other research groups developed a bioresponsive HPMA coating of Ad vector capsids which facilitated an extracellular shielding of vector particles (see Section 4.2), whereas the natural intracellular transport of the vector particles was ensured by shedding of the HPMA shield upon cell entry [84,91]

Thus far, only little is known about bioresponsive PEGylated Ad vectors and their ability to shield against immune and blood components. Espenlaub et al. presented an in vitro laser scanning microscopy assay with fluorescent dyes. In this assay, the PEGs or dyes used were either irreversibly or pH- or redox-sensitively bound to Ad capsids, and their intracellular fate was followed. While irreversibly bound PEGs or dyes coupled to amine groups impaired intracellular trafficking, pH-sensitive hydrazone-bound PEGs had no influence on the intracellular trafficking of the Ad-vectors [98].

The field of bioresponsive PEGylation of Ad vectors is poorly studied especially with regard to shielding against immune and blood components. Bioresponsive PEGylation should efficiently protect Ad vectors extracellularly from host blood components while ensuring vector transport intracellularly and preserving the natural transduction efficiency.

### 5.2. Detargeting and Retargeting with PEGylation

Many research groups used PEGylation strategies not only for detargeting but also for retargeting PEGylated Ad vectors to tissues or cells of choice, such as tumor cells. Tissue- or organ-specific targeting is a major challenge towards Ad vector-mediated gene therapy or virotherapy.

The group of Lanciotti et al. retargeted vectors PEGylated with heterobifunctional T-MPEG-mal coupled with FGF-2 protein to tumor cells via the FGF receptor [119]. In vitro experiments showed high transduction levels in cells of the ovarian cancer cell line SKOV-3, which express the FGF-receptor. Further in vivo experiments showed similar results: vectors were successfully detargeted from off-target organs such as the liver and spleen and retargeted to intraperitoneal tumor cells. The immunological response to PEGylated vectors was decreased, as in vitro; PEGylated vectors were neutralized less; and after treatment of BALB/c mice with PEGylated vectors, lower Th-1 and -2 responses of splenocytes were detected compared to the administration of un-PEGylated vectors [119].

However, PEGylation goes beyond masking vectors from antibodies and immune cells. Hofherr et al. showed that PEGylated vectors reduced the adverse effect of thrombocytopenia and disseminated intravascular coagulation as well as the elevation of D-dimers in mice [120].

As mentioned in the first part of this review (see Section 2.2.2), the idea of the geneti-chemical approach was first established by Kreppel et al. [79,83]. A chemically reactive group is introduced at specific solvent-exposed sites of one or more major capsid proteins via minor genetic modifications. The added reactivity can be further used to specifically mask the mutated protein with PEG moieties. This approach can be used for detargeting as well as for retargeting purposes.

A great example of site-specific PEGylation was achieved with the introduction of a peptide containing a central cysteine residue into the HI loop of fiber. The cysteine -SH groups could be further specifically and covalently attached to mal-PEG. Moreover, this study showed that retargeting of the vector to the transferrin receptor, which is highly expressed in tumor cells, was possible by coupling full-length transferrin to the other end of the heterobifunctional PEG [79].

The introduction of a cysteine residue in HVR5 of hexon, followed by thiol-based coupling with small PEG moieties, prevented the binding of coagulation FX to the viral capsid and showed decreased hepatotropism. The vector was then successfully retargeted to liver cells by chemically coupling transferrin to hexon [80].

In a study of Krutzke et al., first-generation Ad vectors were generated with a mutation in HVR7 and in fiber in order to ablate the vector’s ability to bind to the coagulation FX and CAR receptor, respectively [37]. Another point mutation resulting in a cysteine residue in HVR1 enabled site-specific PEGylation of this HVR. Although FX binding was ablated, hepatocyte transduction by 2 kDa- and 5 kDa-PEGylated Ad-HVR1-ΔCAR-ΔFX was shown in BALB/c mice as well as in antibody-deficient JHD mice. These data indicated an FX and natural antibody-independent mechanism of liver transduction. This study also provided evidence that the specific PEGylation of Ad-HVR1-ΔFX replaced the FX shield and protected the viral particles from natural antibodies and complement factors. Furthermore, PEGylated Ad-HVR1-ΔFX prevented the binding of C3b, thus protecting against opsonization through complement as well as the uptake by macrophages through scavenger receptors [37].

Site-specific mutations and further PEGylation of capsid proteins, particularly hexon, elucidated new capabilities of this specific protein and its significance in the vector’s pathogenicity and biology. Future studies applying or even improving this technique can enlighten the still unknown interactions between humans and Ads and develop new approaches for overcoming major obstacles in Ad gene therapy.

### 5.3. Immune Evasion with PEGylation and PEGylated Ad Vectors as Vaccines

The main goal of capsid shielding is to prevent interactions of vectors with components of the immune system, such as pre-existing neutralizing antibodies, natural IgM, and opsonization, through complement and macrophages. Adenoviral immunity in the human population, due to natural infections, is one of the main obstacles in adeno-based systemic applications. Furthermore, since globally a large fraction of the human population is currently vaccinated with Ad-based vectors, strategies for reapplication of these vectors will be mandatory. Therefore, the need for optimizing PEGylation is highly important, and promising results have been shown from various research groups.

Of great importance in this field are the studies of Croyle et al. [121,122] and, as mentioned above, the studies of O’Riordan et al. [118]. Croyle et al. demonstrated that fully MPEGylated vectors with different reactive groups, such as CC-MPEG, SS-MPEG, and T-MPEG, resulted in reduced cytotoxic lymphocyte responses in vivo. Although shielded vectors evaded humoral and adaptive immune responses, the readministration of the same PEGylated vector showed less efficient gene expression, indicating that natural antibodies against Ad vectors could potentially still become an additional obstacle for Ad gene therapy. However, the researchers furthermore showed that using different activation groups for PEG coupling resulted in the same gene expression levels after readminstration [118,121,122].

Wortmann et al. modified capsids of Ad vectors expressing the hepatitis B surface antigen (HBsAg) with SPA-PEG 20 kDa [123]. Surprisingly, these excessively PEGylated vaccine vectors induced strong anti-transgene product-directed (anti HBsAg) humoral and cellular responses, despite detargeting vectors from CAR. Even in the presence of antibodies against Ad5 after plasma transfer, the PEGylation allowed for evasion from these anti-Ad immunity in vivo. PEGylated vectors when compared to their un-PEGylated counterparts, achieved a significantly higher number of HBsAg CD8^+^/IFN-γ^+^ positive T-cells. These findings were consistent with the results of Mok et al. [97]. In this study, SPA-PEGylated vectors appeared to be less phagocytosed in vitro by Raw264 macrophages, evaluated via qPCR. Furthermore, in vivo analyses regarding liver specific macrophages showed that the abovementioned vectors retained the uptake through hepatocytes but with a decreased uptake by liver specific macrophages like Kupffer cells [97].

An important study by Khare et al. using geneti-chemical shielding revealed more information about the contribution of the different hexon HVRs to the virus’ biology [124]. They introduced cysteine residues in all hexon HVRs and blocked them by PEGylation to investigate the function of this important capsid protein. Transduction in vitro was not affected, but the authors provided evidence that the PEGylation of HVR 1,2,5,7 enhanced liver transduction in mice in conjunction with reduced recognition by Kupffer cells [124].

The immunoevasion potentials of PEGylated vectors for priming and boosting vaccination were also investigated by Weaver and Barry et al. [125]. Ad-immune mice were injected with PEGylated Ads (PEG sizes upt to 35 kDa) intramuscular (IM) or intranasally. Although the vectors initially appeared inactive in vitro, transduction and transgene delivery in vivo were possible, thus confirming the findings by Wortmann et al. [123]. Additional experiments examined the effects of PEGylated vectors after boosting immunization. PEGylated vectors elicited high titers of antibodies against the transgene product similar to those achieved by the unmodified vector after priming, indicating once more that this polymer shield has the ability to medate evasion of the vector particles from neutralizing antibodies. After priming immunization of Ad naïve mice, T-cell responses were also assessed. Intranasally administered 35 kDa PEG-Ad vaccines generated higher levels of neutralizing antibodies when compared to the 5 kDa PEG-Ad. Interestingly, further analyses using 5 kDa PEG-Ad for priming demonstrated that after boosting with either unmodified or modified vectors, strong T-cell responses could be elicited. IM administration of all vectors reached high neutralizing antibody levels, but after boosting vaccination, strong T-cell responses were also achieved. In general, the authors suggested that T-cell and antibody responses could be enhanced after boosting immunization with PEGylated vectors and that priming vaccination with modified vectors is able to induce higher immune responses when compared with unmodified vectors [125].

### 5.4. Preventing Ad-Mediated Toxicity with PEGylation

Ad vectors can be utilized in all fields of virotherapy. They can successfully deliver genes for gene therapy and are able to induce significant immunity against pathogens when used as genetic vaccines. However, the immediate Ad toxicity remains a limiting factor for their use. Cell damage causes not only tissue damage, but in vivo experiments have shown that this cell damage elicited the activation of the complement cascade and therefore further immune responses [126].

The value of PEGylation in reducing tissue-related toxicity was investigated by Mok et al. [97]. In this study, whole capsid SPA-PEGylated first-generation vectors (FG) resulted in low transduction levels in vitro. This was in contrast with in vivo data, which demonstrated similar distribution and transduction levels of the SPA-PEGylated and unmodified vectors. Furthermore, FG-PEGylated vectors resulted in lower interleukine-6 (IL) levels, measured at different time points, both in vivo and in vitro in a macrophage cell line. In particular, IL-6 release was reduced by 10-fold in the first 48 h of infection. Moreover, in mice, the administration of PEGylated and non-PEGylated vectors did not cause an elevation of the liver enzymes alanine aminotransferase (ALT) and aspartate aminotransferase (AST), which are highly released in the case of liver damage, indicating that PEG is not liver toxic.

Interestingly, the researchers also looked into the PEGylation of helper-dependent HD-Ad vectors IL-6 responses and especially liver damage were reduced when using PEGylated HD-vectors suggesting that these vectors could be safer for in vivo administration [97]. Similarly, another study showed that the secretion of other cytokines, such as IL-2, tumor necrosis factor-α, and IL-6, was also significantly lower after vector administration, and Ad-induced thrombocytopenia in mice was not observed [127]. These data underline the safety profile of PEGylated HD-vectors.

## 6. Conclusions

To date, a wide variety of technologies to modify Ad vector capsids has been developed based on scientific knowledge about desirable and undesirable vector–host interactions. Ad vectors are already being successfully used as genetic vaccines; however, their use as classic gene transfer vectors is still hampered because none of the technologies summarized here was sufficient to completely overcome the hurdles imposed by the complex network of vector–host interactions. In particular, the dose-dependent toxicity of systemically delivered Ad vectors and problems arising from pre-existing anti-vector immunity have not been solved so far. Presumably, only a combination of genetics and chemistry might allow for the development of safe and efficient Ad-based vectors in the future.

Genetic modifications typically yield highly defined and uniform particles and can be used to generate vectors detargeted from their primary and secondary receptors. However, for each adenovirus type, the precise sites of receptor interactions and the precise sites involved in unwanted interactions (for example, with blood components) must be identified to the amino acid level prior to successful genetic detargeting. The same holds true for potential insertion sites to retarget the vector particles by peptide ligands. Thus far, insertion sites have been characterized to great detail for Ad5-based vectors, but only very little is known about such sites for rare Ad types. We think that in the near future an in-depth characterization of rare Ad types, including the description of insertion sites/detargeting mutations, will be required to increase the chances for clinically successful Ad vector development. The example of Atasheva et al. [70] discussed above is pioneering for Ad5 and demonstrates that the substantial modulation of Ad vector tropism in vivo is feasible by multiple genetic modifications. However, the problem of adaptive antibody responses remains unsolved.

In addition, it seems likely that in the near future, novel producer cell lines will be required in order to produce such genetically modified de- and retargeted vector particles. It is well known that the generation of cell lines suitable for Ad vector production is anything but trivial, and research projects with a focus on the generation of cell lines for the production of next generation Ad vectors should be fostered not only on an industrial but also on an academic basis.

It is currently unclear if genetic modifications alone will suffice to circumvent the barriers for repeated and systemic Ad vector delivery to patients. Here, chemical modifications offer the possibility of shielding the vector particles. The advantage of shielding by chemical modifications is that conventional producer cell lines can be used, and the sites to be shielded do not need to be described in detail. However, there is a risk that the shielding of larger capsid areas will interfere with biological vector functions, such as intracellular capsid transport and/or nuclear import of the vector DNA. It has become clear in the recent years that dense and tight shielding bears the risk of generating inactive particles that cannot undergo the dynamic structural changes that are required for a successful transduction process. Therefore, bioresponsive shielding modes have been developed that allow a reversible shield to be generated that is removed from the capsid upon cell entry (for example). In addition, a more fine-tuned shielding, such as the thiol-directed geneti-chemical capsid shielding, has been shown to be advantageous, simply due to modifying fewer capsid sites compared to amine-directed large area shielding. However, similar to successful genetic modifications, successful geneti-chemical modifications depend on the precise knowledge of interaction sites on the capsid and might suffer from incomplete shielding against antibodies. We think that special focus should be put on the development of novel bioresponsive coupling modes, which currently seem to be a potential solution for pre-existing humoral immunity. Obviously, both the coupling mode and the nature of the shielding molecule itself are very important. While PEG and HPMA are important examples, novel shielding moieties based, for example, on carbohydrates or even lipids might be developed in the near future.

The last decades of Ad vector development have taught us that there will not be one “magic bullet” that fits all needs. The capsids of second-generation Ad vector-based vaccines to be delivered intramuscularly will likely look different than those of future oncolytic Ads for virotherapy that are delivered intravenously. The further development of existing technologies and novel combinations are most likely to have success in specific applications, may it be in vaccine development, virotherapy or even classic gene therapy.

## Figures and Tables

**Table 1:** Overview of the discussed shielding polymers and their main characteristics. Reactivity of the polymers was directed to amine- (–NH) or thiol- (–SH) groups, whereas the functionality describes the number of reactive groups per polymer (monofunctional = one reactive group; heterobifunctional = two different reactive groups; polyfunctional = many reactive groups). Bioresponsive polymers were coupled reversible (under physiological conditions) to the capsid.

Abbreviation	Polymer	Reactivity	Functionality	Bioresponsive
**ONp-HPMA**	4-nitrophenoxy-poly-*N*-(2-hydroxypropyl) methacrylamide	–NH	polyfunctional	no
**mal-HPMA**	maleimide-poly-*N*-(2-hydroxypropyl) methacrylamide	–SH	polyfunctional	no
**OPSS-HPMA**	orthopyridyl-disulfide-poly-*N*-(2-hydroxypropyl) methacrylamide	–SH	polyfunctional	yes
**Next Generation HPMA**	EC208	–NH	polyfunctional	yes
**T-MPEG**	tresyl-monomethoxypolyethylene glycol	–NH	monofunctional	no
**CC-MPEG**	cyanuric chloride monomethoxypolyethylene glycol	–NH	monofunctional	no
**SS-MPEG**	succinimidyl succinate monomethoxypolyethylene glycol	–NH	monofunctional	no
**T-MPEG-mal**	tresyl-polyethylene-glycol-maleimide	–NH, –SH	heterobifunctional	no
**mal-PEG**	maleimide-polyethylene glycol	–SH	monofunctional	no
**SPA-PEG**	succinimidyl propionate polyethylene glycol	–NH	monofunctional	no

## Data Availability

Not applicable.
